# Evaluation and prioritization of food environment policies in Norway using the Healthy Food Environment Policy Index (Food-EPI)

**DOI:** 10.29219/fnr.v67.9117

**Published:** 2023-04-17

**Authors:** Liv Elin Torheim, Anne Lene Løvhaug, Camilla Sanne Huseby, Sigrun Henjum, Laura Terragni, Maartje Poelman, Janas Harrington, Stefanie Vandevijvere, Gun Roos, PEN consortium

**Affiliations:** 1 Department of Nursing and Health Promotion, OsloMet – Oslo Metropolitan University; 2 Chair group Consumption and Healthy Lifestyles, Wageningen University, Wageningen, The Netherlands; 3 HRB Centre for Health and Diet Research, School of Public Health, University College Cork, Ireland; 4 Sciensano, Department of epidemiology and public health, Brussel, Belgium; 5 Consumption Research Norway – SIFO, OsloMet – Oslo Metropolitan University

**Keywords:** food environment, public health, policy, non-communicable diseases, benchmarking

## Abstract

**Background:**

Government policies promoting healthier food environments can contribute to healthier diets and prevent obesity and diet-related non-communicable diseases.

**Objective:**

To assess the level of implementation of internationally recommended food environment policies in Norway and establish prioritised actions to create healthier food environments.

**Design:**

The Healthy Food Environment Policy Index (Food-EPI) was adapted to the Norwegian context. It comprised 45 good practice indicators of government food environment policy and infrastructure support. Systematically compiled evidence of relevant policies was verified by government officials and formed the basis for assessing the level of implementation of these policies compared against international best practice benchmarks. The assessment was done by a national non-government expert panel (*n* = 35). The experts thereafter proposed and prioritized policy actions for government implementation.

**Results:**

Most indicators were rated at a medium or high degree of implementation in both the policy action (69%) and the infrastructure support (77%) components. No indicators were rated as having ‘none or very little implementation’. Among the 14 recommended policy actions, active use of price regulation to increase the price of unhealthy foods and decrease the price of healthy foods was the highest priority. Other top priorities were ensuring healthy food environments in public settings and introducing free school meals. Demonstrating knowledge-based and coherent political leadership in public health nutrition policies was the highest priority among the 11 recommended infrastructure support actions.

**Conclusion:**

The overall policies in Norway to promote a healthy diet show a medium to high level of implementation. This study highlights that there is still room for additional improvements in Norwegian policies and infrastructure support to promote healthy food environments.

## Popular scientific summary

- Overweight, obesity and diet-related NCDs are increasing globally, including in Norway, driven by food environments that promote an unhealthy diet.
- We assessed whether recommended policies and actions to create healthy food environments are implemented in Norway.
- Norway is doing better than many other countries, however, there are still many recommended policies that could be implemented to support healthier food environments, e.g. price regulation, school meals and clearer political leadership for improved nutrition.

Globally, obesity is increasing at an alarming rate (1) and unhealthy diets are a major contributor to the loss of healthy life years (2). This is the case also in Norway where the current rate of overweight including obesity is about 77% among men and 58% among women, and the obesity rate is about 26% (3). This leads to an increased risk of non-communicable diseases (NCDs) and has large consequences at the individual and societal levels, including health spending (4).

Food environments, defined as ‘the collective physical, economic, policy and socio-cultural surroundings, opportunities and conditions that influence people’s food and beverage choices and nutritional status’ (5), are central in shaping peoples’ dietary intake (6). Unhealthy food environments play an important role in the global increase in overweight, obesity and nutrition-related NCDs (5).

Norway has implemented public health nutrition policies since the 1970s. It was one of the first countries to introduce taxes on confectionary and non-alcoholic beverages (7). The government has enforced a regulation that prohibits any marketing to children in broadcast media (8), and it also supports an industry-led self-regulation scheme that limits the marketing of unhealthy foods to children (9). The Nordic Keyhole, a public, voluntary front-of-pack labelling scheme that can only be used on foods meeting certain nutritional criteria, has been used in Norway since 2009 (10). In recent years, two subsequent governmental action plans for healthier diets have set targets to change the diet in line with the national dietary guidelines and to reduce social inequalities in diet (11, 12). Collaboration with the food industry is an important strategy in both action plans, and a formalized public–private partnership between the Norwegian health authorities and the food industry was signed in 2016 and is planned to run until 2025. The partnership has set targets for reduced intake of salt, sugar and saturated fat, and for increased intake of fruit and vegetables, whole-grain foods, and fish and seafood in the population (13). The main strategy for achieving the targets is product reformulation.

There have been several improvements in the diet in Norway, with increased intake of fruits and vegetables (observed over several decades) and a reduced intake of added sugars (observed for the last 20 years). However, the intake of salt, sugar, saturated fat and red and processed meat is still above the recommended intake, and the intake of fruits, vegetables, fish and whole grains is still below the recommendations (14).

An important contribution to determine how Norway can step up the actions to improve food environments is to assess the level and range of implemented policy actions. A tool for doing such an analysis is provided through the Healthy Food Environment Policy Index (Food-EPI) (15), which was developed by the International Network for Food and Obesity/NCDs Research, Monitoring and Action Support (INFORMAS) (5, 15). INFORMAS is a global network of researchers and public interest organisations that aims to monitor and benchmark public and private sector actions to support healthy food environments and reduce obesity and NCDs. By assessing government policy actions rather than risk factors or health outcomes, the Food-EPI complements the WHO NCD Global Monitoring Framework (16). It is assumed that Food-EPI policy monitoring can stimulate governmental enhanced action to improve the healthiness of food environments (5). The Food-EPI process has been implemented in several countries, e.g. in Australia, New Zealand, and several Asian, Latin-American and African countries, in addition to Canada and the UK (17). As part of the Policy Evaluation Network (PEN), a project funded through the European Union’s (EU) Joint Programming Initiative ‘A Healthy Diet for a Healthy Life’ (JPI HDHL), five European countries set out to implement the Food-EPI to evaluate the food environment policies in these countries, of which Norway was one (18). In addition, six other European countries are undertaking Food-EPI studies under the EU Horizon2020 Science and Technology in child Obesity Policy Project (STOP) (19).

Our aims were to (1) determine the degree of implementation of recommended food environment policies and infrastructure support by the Norwegian Government, against international benchmarks and (2) establish prioritized recommendations for the government based on identified implementation gaps.

## Methods

### The Food-EPI framework: tool and process

The Food-EPI tool (15, 20) covers governmental measures according to two components: ‘policy’ and ‘infrastructure support’. The policy component represents internationally recommended policies for enabling healthy food environments. It includes seven policy domains (food composition, food labelling, food promotion, food prices, food provision, food retail, and food trade and investment). The infrastructure support component reflects systems that facilitate policy development and good nutrition governance. It includes six infrastructure support domains (leadership, governance, monitoring and intelligence, funding and resources, platforms for interaction, and health-in-all-policies). Each policy and infrastructure support domain consists of two to five good practice indicators that represent distinct policies. The indicators are formulated as ideal good practice statements, based on recommended policies (e.g. one indicator under the food promotion policy domain is formulated as: *Effective policies are implemented by the government to restrict exposure and power of promotion of unhealthy foods to children through broadcast media (TV, radio)*)*.* A set of benchmarks has been established for each indicator. These are examples of real-world government actions that are collated by the INFORMAS team and considered ‘best practice’ (e.g. for the policy indicator on food promotion there are four international benchmarks, describing regulations and policies to restrict food promotion in broadcast media that are implemented in Quebec (Canada), Norway, Ireland, and Chile).

**Fig. 1:**
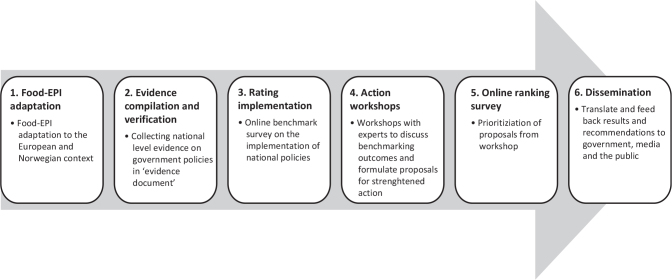
Steps in the Food Environment Policy Index (Food-EPI) process in Norway. Adapted from Swinburn et al. (15).

The Food-EPI **process** (15, 20), leads to (1) an assessment and benchmarking of actual policies in a specific country. In this process, a panel of national public health and nutrition experts rates the policies the government is implementing according to the Food-EPI indicators compared with international benchmarks (steps 1-3, Fig 1).

The Food-EPI process also leads to (2) a formulation of actions that the experts recommend should be implemented to improve food environments in the country, and a prioritization of what they find most important (steps 4-5, Fig. 1). The Food EPI process includes also dissemination of results and recommendations to the government, media, and public (step 6).

In the following, we describe how the Food-EPI tool and process have been adapted and conducted in Norway as part of the PEN project.

In **Step 1**, the domains and indicators were adapted to the European and Norwegian contexts. In the process of adapting Food-EPI in Norway and the other European countries in the PEN, the policy domain ‘Food trade and investment’ was excluded, since the European countries, in general, are bound by international trade agreements, with little leeway at the national level. In Norway, three additional indicators were excluded: one indicator assessing the existence of food-related income support programs for healthy foods since Norwegian income support programs are not earmarked for food support and two infrastructure support indicators assessing budget to nutrition and financing of research regarding obesity and NCDs since it was challenging to identify these budgets. The Norwegian Food-EPI thus consisted of six policy domains and six infrastructure support domains with 23 and 22 indicators, respectively.

In **Step 2**, the Norwegian research team collected evidence of policy implementation that was in place at the time of data collection (autumn 2019) in Norway for each indicator. The information was mainly collected through Internet searches of governmental documents and assembled in an ‘evidence document’. The completeness and accuracy of the evidence were verified by relevant government officials.

The evidence document also contained benchmarks, i.e. examples of international best practices, for each indicator. These had initially been developed through the INFORMAS project (21) and were updated with new examples through thorough discussions among the participating researchers in the PEN project (22).

In **Step 3**, a national expert panel was established to rate the extent of implementation against international benchmarks and to identify concrete actions to create healthy food environments. Recruitment of the experts was based on professional competence in public health nutrition or public health. Individuals with affiliations to the food industry or to governmental bodies under scrutiny (e.g. the Ministry of Health or the Directorate of Health) were purposefully not invited to avoid conflicts of interest, in line with the Food-EPI protocol. Recruitment started based on the research group’s network and was extended by an open invitation given in an information seminar and covered by a Norwegian nutrition journal, in May 2019 (23). In total, eighty independent experts were either contacted directly (*n* = 77) or responded to the open invitation (*n* = 3). Of these, 38 agreed to participate (*n* = 24 from academic institutions, *n* = 5 from NGOs, *n* = 5 from other civil society organisations, and *n* = 4 from other types of organisations or from municipalities). All interested experts submitted a conflict of interest form that was assessed by the research team.

The benchmarking exercise was performed using the online survey tool Nettskjema (24). The experts received the evidence document in January 2020 and were instructed to use the information provided to rate the degree of implementation of the Norwegian government’s policies for each indicator as compared with the international benchmarks, using a Likert scale of 1 to 5 (1 ≤ 20% implemented, 2 = 20–40% implemented, 3 = 40–60% implemented, 4 = 60–80% implemented, 5 = 80–100% implemented). There was also a ‘cannot rate’ option. Experts were asked to consider the various steps of the ‘policy cycle’ (agenda-setting and initiation, policy development, implementation, and evaluation) in their ratings (20).

In **Step 4**, a full-day in-person workshop was conducted in Oslo (February 2020) to identify proposals for recommended policy actions for healthier food environments in Norway. The participating experts were presented with the rating results from the online survey. Based on identified policy gaps and considering the Norwegian context, the experts proposed measures, which were voted on during the workshop; only proposals that received support from at least 50% of the participants were taken further and refined and overlapping proposals were merged.

Following the workshop, in **step 5**, members of the expert panel were invited to participate in an online ranking of the proposed measures. The experts were asked to rank the policy and infrastructure support proposals according to two different and separate criteria: how *important* the measure is and how likely it is that the measure will be implemented (*achievability*). The proposals in the policy component should also be ranked according to a third criterion: to what extent experts believed the measure can contribute to *reducing social inequality* in dietary intake. This criterion was introduced in the PEN project to better integrate equality in the Food-EPI process.

In **step 6**, the results from the benchmarking process and the recommendations for strengthened action were assembled in a report (25) and disseminated to interested stakeholders, media and policymakers through an open, digital launch seminar conducted in September 2020.

### Evaluation of the process

All members of the expert panel were invited to fill in an evaluation form regarding their participation in the project. It included an evaluation of both the Food-EPI tool and the process using five-point Likert scales.

### Data analyses

The mean rating scores for each indicator were categorized into four implementation levels: high (>75% implemented), medium (51–75% implemented), low (26–50 % implemented), and none or very little implementation (<25% implemented) against international benchmarks. Assessment of inter-rater reliability (IRR) using the Gwet AC2 statistic (Agreestat 2013.1, Advanced Analytics, Gaithersburg, USA) was performed to measure the degree to which the expert panel members agreed in their assessment of each of the indicators. The prioritizing scores were summed for each criterion (importance, achievability and potential to level out social inequality (only in the policy component)) for each proposal. Within each of the two components (‘policy’ and ‘infrastructure support’), the proposed actions were ranked according to the sum of the prioritization scores. The ranking according to each separate criterion was also examined. Descriptive analysis (mean and percentages) was used to present the rating within each domain.

### Ethical approval

The study protocol was approved by the Norwegian Centre for Research Data (ID 355179). All participants gave their written informed consent for inclusion before they participated in the study.

## Results

Thirty-five experts participated in at least one of the activities: 34 in the online benchmarking exercise, 19 in the face-to-face workshop (including one expert who had not taken part in the online benchmarking), and 21 provided feedback to the online prioritization, leading to response rates of 42%, 24% and 26%, respectively.

### The extent of policy implementation and infrastructure support in Norway against international benchmarks

**Fig. 2:**
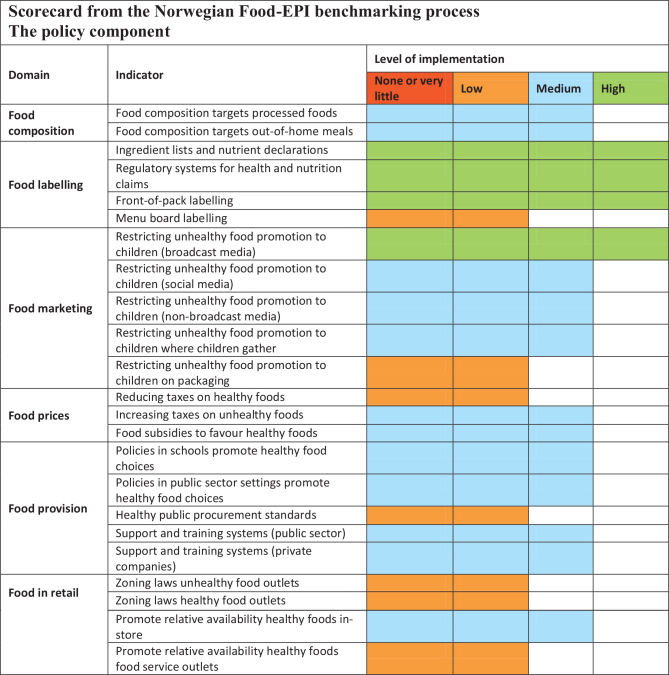
Ratings of the level of implementation compared with international best practice for 23 Food Environment Policy Index (Food-EPI) indicators within 6 policy domains. High (>75% implemented), medium (51–75% implemented), low (26–50% implemented), and none or very little (<25% implemented).

Within the policy component of the Food-EPI (Fig. 2), four indicators (17%) were rated as ‘high’ implementation: ‘ingredient lists and nutrient declarations’, ‘regulatory systems for health and nutrition claims’, ‘front-of-pack labelling’, and ‘restricting unhealthy food promotion to children (broadcast media)’ (94%, 84%, 78% and 85% implementation, respectively). Seven indicators (30%) were rated as ‘low’ implementation, and the three indicators with the lowest rating were ‘menu board labelling’, ‘reducing taxes on healthy foods’, and ‘zoning law for healthy food in outlets’ (28%, 39% and 39% implementation, respectively).

**Fig. 3:**
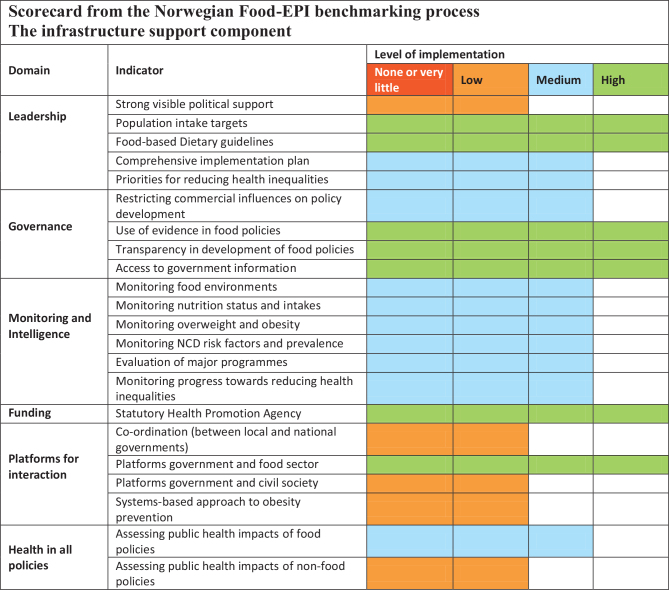
Ratings of the level of implementation compared with international best practice for 22 Food Environment Policy Index (Food-EPI) indicators within six infrastructure domains. High (>75% implemented), medium (51–75% implemented), low (26–50 % implemented), and none or very little (<25% implemented).

Seven out of 22 indicators (32%) in the ‘infrastructure’ component (Fig. 3) were rated as ‘high’ implementation, and the top three were all subdomains within the ‘Governance’ domain: ‘use of evidence in food policies’, ‘transparency in the development of food policies’, and ‘access to government information’ (92%, 95% and 96% implementation, respectively). Five indicators (23%) were rated as ‘low’ implementation with the lowest rating for ‘strong visible political support’, ‘platform for interaction between government and civil society’, and ‘system-based approach to obesity prevention’ (47%, 34% and 28% implementation, respectively). The IRR of ratings performed by the experts was 0.37 (95 % CI 0.28, 0.46).

### Identifying and prioritizing policy actions

During the workshop, a total of almost 60 proposals were initially suggested. After voting over and merging overlapping proposals, the final list consisted of 14 proposals under the policy component and 11 under the infrastructure component (see Supplementary Tables 1 and 2). After the prioritization exercise, the top three recommendations in the policy component were to (1) actively use price policies to shift consumption from unhealthy to healthy foods; (2) ensure healthy food environments in public settings; and (3) introduce free school meals (Table 1). This final prioritisation echoes the ranking list based on the criteria *importance* and potential to *reduce social inequality* but deviates in several ways from the criteria *achievability* (Supplementary Table 1). Most of the recommended policy actions had been rated as either low or medium level of current implementation in the benchmarking process.

**Table 1:** Norwegian Food-EPI expert panels’ top five proposals for government policy action

No.	Top five proposals for policy action	Domain	Average rating of current policies^a^
1	Actively use fiscal policies to shift consumption from unhealthy to healthy foods. This includes to: Introduce a differentiated sugar tax aiming at reducing consumption of sugary foods and drinks Investigate the possibility of introducing taxes to reflect climate/environmental footprint.	Price	Low/ Medium
2	Step up efforts to create healthy food environments and make healthy choices easy in public settings. This includes to: Impose requirements on municipalities for healthy food environments in kindergartens and schools, based on available evidence. The requirement must include that municipalities develop an implementation plan for the use and compliance with national guidelines for food and meals in schools and kindergartens, including school canteens and kiosks. Set requirements for the food offered in public contexts to follow the national dietary guidelines. Set nutritional requirements for the contents of vending machines in public areas.	Provision	Medium
3	Order all municipalities to offer a simple school meal (which at least consists of free school fruit), with room for local adaptation and with state part-financing.	Provision	Medium
4	Demand clearer results in the ongoing public–private partnership (letter of intent with the food sector) to achieve the goals set in the agreement and make food stores healthier. This includes to: Press to set standards for the reduction of saturated fat and sugar in the letter of intent. Consider introducing and publishing a ‘ranking’ of the best and worst actors in the food sector when it comes to nutrient composition in foods, especially regarding salt, sugar, and saturated fats.	Retail	Medium
5	Introduce a legal regulation of the marketing of unhealthy food and drink targeting children. Or, alternatively put pressure on the industry so that the guidelines in the Food Industry Professional Committee (MFU) become stricter than today and to a greater extent in accordance with WHO recommendations. The latter will involve a re-assessment of the exceptions in the MFU guidelines regarding packaging, placement in supermarkets, and sponsorship.	Promotion	Medium/Low

a Reflecting the expert panel’s rating of the indicators in the evidence document

The top three recommendations in the infrastructure support component were to (1) demonstrate clear, knowledge-based and coherent political leadership in public health and nutrition policies; (2) ensure access to qualified nutrition and food competence in the public sector; and (3) ensure strengthening of nutrition as part of public health actions and implementation of ‘health in all policies’ at all levels (Table 2). This list is similar to the ranking based on the criterion *importance* but deviates to a larger extent from the ranking based on *achievability* (Supplementary Table 2). All the recommended infrastructure support actions were related to indicators that had been rated as either low or medium level of current implementation.

**Table 2:** Norwegian Food-EPI expert panels’ top five recommendations for government infrastructure support action

No.	Top five proposals for action within infrastructure support	Domain	Average rating of current policies^a^
1	Demonstrate clear, knowledge-based, and coherent political leadership in public health and nutrition policies This includes to: Strengthen and coordinate public health policy by following up the current action plans on diet and on physical activity and putting in place an NCD strategy. Plan long-term (> 10 years) follow-up of an action plan for a better diet, where: focus areas and measures correspond to goals defined responsibilities at the community level a budget is included to ensure implementation	Leadership	Low
2	Ensure that there is access to qualified nutrition and food competence in the public sector. This means that the authorities should: Introduce requirements for competence for teachers in the Food and Health subject Require municipalities to have staff with relevant nutrition expertise. The competence requirement will vary with the degree of responsibility and level Create positions for both public health nutritionists (for health promotion/disease prevention activities) and for dieticians (clinical nutrition work) with requirements for higher education in nutrition	Funding and resources	*Not assessed*
3	Ensure that nutrition is strengthened as part of public health actions and that ‘health in all policies’ is implemented at all levels. This includes to: Instruct health authorities at all levels to carry out health impact assessments of all policies that may have consequences for the food environment and the population’s nutrition/diet, and develop suitable tools for this Give county governors and municipalities clearer letters of assignment expectations and requirements related to working and reporting on nutrition	Health in all policies	Low
4	Monitor the compliance with the national Norwegian Guidelines for Food and Meals in schools, kindergartens, and after-school clubs, including in school canteens and kiosks.	Monitoring and intelligence	Medium
5	Ensure long-term financing of effective and health promoting nutrition and public health work in counties and municipalities. This includes to: ensure financing of targeted nutrition interventions toward lower socio-economic groups, including evaluation of the interventions earmark funding for health promoting activities in schools and Kindergartens	Funding and resources	*Not assessed*

a Reflecting the expert panel’s rating of the indicators in the evidence document

### Dissemination

The report from the Norwegian Food-EPI project was published online (https://www.jpi-pen.eu/images/reports/FoodEPI-Report-Norway-2020.pdf) and launched in an open webinar in September 2020 attended by around 150 persons. Following the webinar, the Food-EPI project was featured in several national broadcast and newspaper media (e.g. [26, 27]).

### Evaluation of the process

Fourteen out of the 35 members of the expert panel responded to the evaluation survey (response rate of 40%). Of these, all agreed that the Food-EPI framework with its indicators was comprehensive or sufficiently comprehensive. Most of the respondents (*n* = 13) found the three-step process ‘appropriate’ or ‘very appropriate’, but most also (*n* = 9) found it ‘somewhat hard’ to rate Norwegian policies toward international benchmarks. Most (*n* = 12) agreed that Food-EPI could influence nutrition policy positively and all agreed that the Food-EPI project should be repeated to monitor nutrition policy in Norway.

## Discussion

Norway is a country with a high proportion of the population having overweight or obesity (around 67%) and is off track to meeting its targets to stop the increases in diabetes and obesity in line with international commitments (3). We used the Food-EPI to appraise the Norwegian government’s efforts to create healthier food environments by benchmarking their policies and infrastructure support against international best practices (5). The results showed that overall, 24% of the indicators were rated as having high and 49% were rated as having medium implementation. Almost one in three indicators (27% across policy and infrastructure domains) were rated as low implementation, but none were rated at the lowest level. This is a better rating than most other countries that have conducted the Food-EPI process. A pooled level analysis across the eleven European countries having conducted the Food-EPI as part of either the PEN or the STOP projects showed that Finland had the highest proportion of food environment policies rated as “high” or “medium” level of implementation, followed by Portugal and Norway (22). A previous study compared the results of 11 non-European countries that had undertaken Food-EPI studies between 2015 and 2018 (21). Chile had the highest rating and was similar to Norway, with 20% of the indicators rated as high and 40% rated at a medium level of implementation (21). It is, however, important to keep in mind that the benchmarking in Food-EPI is done against examples of international best practices and not against the recommended policies for each good practice indicator (15, 21), which would imply a much higher standard for most indicators.

Within the policy component, four indicators received ‘high’ implementation scores. One of these indicators pertains to restrictions on food marketing aimed at children in broadcast media where Norway has a strict Broadcast Act and is listed as a Food-EPI benchmark for this indicator.

Regarding the indicator for front-of-pack labelling, the experts considered the Nordic Keyhole on a level with the international benchmarks (i.e. the UK traffic light, the Australian Health Star Rating, and the Nutri-Score). Interestingly, this assessment of the Keyhole scheme appears to conflict with the academic literature, since front-of-pack labelling that can be used across all food groups are often considered better tools for consumers compared with endorsement logos (like the Keyhole) that can only be applied to foods that meet certain criteria (28).

Expert recommendations for strengthened government policy action identified school meal policies, taxes on unhealthy foods, and restrictions on food marketing to children (beyond broadcast media) as prioritized areas for strengthened policy action. These prioritizations reflect recommendations from other countries that have undertaken Food-EPI studies (21, 22), are in line with WHO’s ‘best buys’ for NCD prevention policies (29) and are also currently on the Norwegian political public health agenda. For instance, school meal policies have long been on the policy agenda in Norway and the current Norwegian government state that they will introduce school meals during their governance period (30).

In terms of infrastructure support indicators, the expert panel rated seven indicators (32%) as having ‘high’ implementation against international benchmarks, which is a high proportion compared with other countries (21, 22). One of these, ‘Platforms for interaction with the food sector’ has been a prioritized strategy for the Norwegian government for several years, reflected in the public–private partnership between the health authorities and the food sector (13). There are no similar platforms for interaction with civil society and other public health actors, or for within-government coordination, in Norway. The expert panel recommended the establishment of platforms for interaction in these dimensions. Such platforms may help put public health nutrition on the policy agenda and ensure the involvement of civil society and of other government sectors.

Perhaps the most notable ‘low’ score was given to the indicator ‘Strong visible political support’, which is meant to reflect support for nutrition action and NCD prevention at the highest political level. Whereas cooperation with the food industry has been an important strategy for nutrition policy in Norway in the last decade (11), action to reduce diet-related NCDs through other measures has not been prioritized to the same extent. Correspondingly, the expert panel recommended to ’demonstrate clear, knowledge-based and coherent political leadership in public health and nutrition policies’. This included a recommendation to strengthen the coherence between nutrition goals and measures taken, and a recommendation to include long-term budgets to fund policy implementation. An independent third-party evaluation of the current action plan on nutrition came to similar conclusions as it observed a lack of coherence between the plan’s targets and measures as well as inadequate funding of the measures (31). This emphasizes the need for stronger public health and nutrition commitment in Norway, to build upon and strengthen current policies for healthy food environments. Increased funding was also among the top recommended actions in the 11 European countries that have conducted the Food-EPI (22).

### Strengths and limitations

In this study, we used an internationally developed and acknowledged approach that allows for a structured assessment of recommended food environment policies and enables comparison with other countries and adapted the tool to the Norwegian context.

The overall response rate was 42%, and most of the experts participated in the online benchmarking exercise (35 persons). Only 19 persons participated in the one-day prioritization workshop, which could reflect busy schedules and challenges relating to spending a whole day. For those not residing in or close to Oslo, travelling represented another burden in terms of time and costs although the project covered travelling costs. Another reason for the low participation in the workshop could have been the Covid-19 pandemic. Norway implemented travel restrictions on the 12^th^ of March 2020, but some could have taken precautions already at the time of the workshop. Although all 35 members of the expert panel were invited to provide feedback on the online prioritization, only 21 persons did so. The participation rates were similar in the other European countries conducting the Food-EPI as part of the PEN and the STOP projects (22). Ways to increase the participation rate throughout the Food-EPI process should be explored to improve the representativeness of the results.

As opposed to other studies that have implemented the Food-EPI, the IRR score in this study was relatively low (GwetAC2 = 0.37), reflecting a lack of consistency in assessments between participants. The study that compared results from Food-EPI studies undertaken in 11 non-European countries reported higher IRR (GwetAC2 = 0.6-0.8) (21). In the pooled study from 11 European countries, some countries had lower IRR whereas most had similar or higher values (22). In the evaluation, many participants reported that they found the benchmarking difficult, which has been noticed as a limitation of the Food-EPI tool in previous assessments (21). In future use of the Food-EPI process, this should be further investigated.

## Conclusion

This study highlights that there is room for improvement in the Norwegian food environment policies and the infrastructure support systems, although the overall level of implementation is medium to high. The Norwegian expert panel recommended priority actions that may if implemented, help to make food environments healthier and improve the population’s diet. The framework proved useful for placing food environment policies on the public agenda. Comparing the achievements with those reached in other countries can contribute to strengthened government accountability. Tools such as the Food-EPI can contribute to monitoring the Government’s performance for healthier food environments.

## Supplementary Material

Evaluation and prioritization of food environment policies in Norway using the Healthy Food Environment Policy Index (Food-EPI)

## Figures and Tables

**Table:** 

EU	European Union
Food-EPI	The Healthy Food Environment Policy Index
INFORMAS	International Network for Food and Obesity/NCDs Research, Monitoring and Action Support
IRR	Inter-rater reliability
NCD	Non-communicable diseases
PEN	Policy Evaluation Network
WHO	World Health Organization
