# Supplementary material for: Evaluation and prioritization of food environment policies in Norway using the Healthy Food Environment Policy Index (Food-EPI)
Source: Food Nutr Res. 2023 Apr 17;67:10.29219/fnr.v67.9117. doi: 10.29219/fnr.v67.9117 (PMC11801381; doi:10.29219/fnr.v67.9117)
Supplement: Evaluation and prioritization of food environment policies in Norway using the Healthy Food Environment Policy Index (Food-EPI) [file FNR-67-9117-S001.docx]

**Supplementary Tables**

**Supplementary Table 1 - Recommended actions in the Policy Component**

Table S1-A – Recommended policy actions: ranking based on the combination of the criteria *importance*, *achievability* and *potential to reduce social inequality*

| # | Recommended action | Corresponding Food-EPI domain |
| --- | --- | --- |
| 1 | Actively use fiscal policies to shift consumption from unhealthy to healthy foods. | PRICES |
| 2 | Step up efforts to create healthy food environments and make healthy choices easy in public settings. | PROVISION |
| 3 | Order all municipalities to offer a simple school meal (which at least consists of free school fruit), with room for local adaptation and with state part-financing. | PROVISION |
| 4 | Demand clearer results in the ongoing public-private partnership (Letter of Intent with the food sector) to achieve the goals set in the agreement and make food stores healthier. | RETAIL |
| 5 | Introduce legal regulations of the marketing of unhealthy food and drink targeting children. | PROMOTION |
| 6 | Set clearer requirements for municipalities' use and compliance with advice and guides under the Public Health Act and the Planning and Building Act regarding diet and nutrition, to promote healthy food environments in local communities. | RETAIL |
| 7 | Work to promote healthy in-store food environments in dialogue with the food industry | RETAIL |
| 8 | Ensure that kindergarten children are protected from advertising through an amendment and clarification in the Kindergarten Act | PROMOTION |
| 9 | Work to promote healthy food environments in local communities in dialogue with the food industry and encourage the actors to introduce "safety zones" around schools, sports arenas, and the like to reduce the availability of unhealthy foods. | RETAIL |
| 10 | Introduce legislation regulating the sale and marketing of high-caffeinated energy drinks to protect children and young people. | PROMOTION |
| 11 | Introduce a mandatory labelling scheme for calories, fats, salt, and sugar in the food service restaurants | LABELLING |
| 12 | Initiate earmarked investment in research in agriculture to stimulate increased production of plant-based food. | PRICES |
| 13 | Be an active driving force towards the EU to change relevant parts of the food legislation that Norway is obliged to follow through the EEA agreement | LABELLING |
| 14 | Include health and environmental considerations in the purpose of the regulations for the Sales and Marketing Council (an independents council aiming at regulating the Norwegian market for various agricultural products) | PRICES |

Table S1-B – Recommended policy actions: ranking based on the criteria *importance*

| # | Recommended action | Corresponding Food-EPI domain |
| --- | --- | --- |
| 1 | Actively use fiscal policies to shift consumption from unhealthy to healthy foods. | PRICES |
| 2 | Step up efforts to create healthy food environments and make healthy choices easy in public settings. | PROVISION |
| 3 | Demand clearer results in the ongoing public-private partnership (Letter of Intent with the food sector) to achieve the goals set in the agreement and make food stores healthier. | RETAIL |
| 4 | Order all municipalities to offer a simple school meal (which at least consists of free school fruit), with room for local adaptation and with state part-financing. | PROVISION |
| 5 | Introduce legal regulations of the marketing of unhealthy food and drink targeting children. | PROMOTION |
| 6 | Work to promote healthy in-store food environments in dialogue with the food industry | RETAIL |
| 7 | Set clearer requirements for municipalities' use and compliance with advice and guides under the Public Health Act and the Planning and Building Act regarding to diet and nutrition, to promote healthy food environments in local communities. | RETAIL |
| 8 | Work to promote healthy food environments in local communities in dialogue with the food industry and encourage the actors to introduce "safety zones" around schools, sports arenas, and the like to reduce the availability of unhealthy foods. | RETAIL |
| 9 | Introduce a mandatory labelling scheme for calories, fats, salt, and sugar in the food service restaurants | LABELLING |
| 10 | Introduce legislation regulating the sale and marketing of high-caffeinated energy drinks to protect children and young people. | PROMOTION |
| 11 | Be an active driving force towards the EU to change relevant parts of the food legislation that Norway is obliged to follow through the EEA agreement | LABELLING |
| 12 | Ensure that kindergarten children are protected from advertising through an amendment and clarification in the Kindergarten Act | PROMOTION |
| 13 | Initiate earmarked investment in research in agriculture to stimulate increased production of plant-based food. | PRICES |
| 14 | Include health and environmental considerations in the purpose of the regulations for the Sales and Marketing Council (an independents council aiming at regulating the Norwegian market for various agricultural products) | PRICES |

Table S1-C – Recommended policy actions: ranking based on the criteria *achievability*

| # | Recommended action | Corresponding Food-EPI domain |
| --- | --- | --- |
| 1 | Ensure that kindergarten children are protected from advertising through an amendment and clarification in the Kindergarten Act | PROMOTION |
| 2 | Set clearer requirements for municipalities' use and compliance with advice and guides under the Public Health Act and the Planning and Building Act regarding diet and nutrition, to promote healthy food environments in local communities. | RETAIL |
| 3 | Demand clearer results in the ongoing public-private partnership (Letter of Intent with the food sector) to achieve the goals set in the agreement and make food stores healthier. | RETAIL |
| 4 | Actively use fiscal policies to shift consumption from unhealthy to healthy foods. | PRICES |
| 5 | Initiate earmarked investment in research in agriculture to stimulate increased production of plant-based food. | PRICES |
| 6 | Work to promote healthy in-store food environments in dialogue with the food industry | RETAIL |
| 7 | Introduce legislation regulating the sale and marketing of high-caffeinated energy drinks to protect children and young people. | PROMOTION |
| 8 | Introduce legal regulations of the marketing of unhealthy food and drink targeting children. | PROMOTION |
| 9 | Step up efforts to create healthy food environments and make healthy choices easy in public settings. | PROVISION |
| 10 | Include health and environmental considerations in the purpose of the regulations for the Sales and Marketing Council (an independents council aiming at regulating the Norwegian market for various agricultural products) | PRICES |
| 11 | Order all municipalities to offer a simple school meal (which at least consists of free school fruit), with room for local adaptation and with state part-financing. | PROVISION |
| 12 | Introduce a mandatory labelling scheme for calories, fats, salt, and sugar in the food service restaurants | LABELLING |
| 13 | Work to promote healthy food environments in local communities in dialogue with the food industry and encourage the actors to introduce "safety zones" around schools, sports arenas, and the like to reduce the availability of unhealthy foods. | RETAIL |
| 14 | Be an active driving force towards the EU to change relevant parts of the food legislation that Norway is obliged to follow through the EEA agreement | LABELLING |

Table S1-B – Recommended policy actions: ranking based on the criteria *potential to reduce social inequality*

| # | Recommended action | Corresponding Food-EPI domain |
| --- | --- | --- |
| 1 | Actively use fiscal policies to shift consumption from unhealthy to healthy foods. | PRICES |
| 2 | Order all municipalities to offer a simple school meal (which at least consists of free school fruit), with room for local adaptation and with state part-financing. | PROVISION |
| 3 | Step up efforts to create healthy food environments and make healthy choices easy in public settings. | PROVISION |
| 4 | Work to promote healthy in-store food environments in dialogue with the food industry | RETAIL |
| 5 | Introduce legal regulations of the marketing of unhealthy food and drink targeting children. | PROMOTION |
| 6 | Set clearer requirements for municipalities' use and compliance with advice and guides under the Public Health Act and the Planning and Building Act regarding diet and nutrition, to promote healthy food environments in local communities. | RETAIL |
| 7 | Demand clearer results in the ongoing public-private partnership (Letter of Intent with the food sector) to achieve the goals set in the agreement and make food stores healthier. | RETAIL |
| 8 | Work to promote healthy food environments in local communities in dialogue with the food industry and encourage the actors to introduce "safety zones" around schools, sports arenas, and the like to reduce the availability of unhealthy foods. | RETAIL |
| 9 | Ensure that kindergarten children are protected from advertising through an amendment and clarification in the Kindergarten Act | PROMOTION |
| 10 | Introduce legislation regulating the sale and marketing of high-caffeinated energy drinks to protect children and young people. | PROMOTION |
| 11 | Introduce a mandatory labelling scheme for calories, fats, salt, and sugar in the food service restaurants | LABELLING |
| 12 | Be an active driving force towards the EU to change relevant parts of the food legislation that Norway is obliged to follow through the EEA agreement | LABELLING |
| 13 | Include health and environmental considerations in the purpose of the regulations for the Sales and Marketing Council (an independents council aiming at regulating the Norwegian market for various agricultural products) | PRICES |
| 14 | Initiate earmarked investment in research in agriculture to stimulate increased production of plant-based food. | PRICES |

**Supplementary Table 2 - Recommended actions in the Infrastructure Support Component**

Table S2-A – Recommended infrastructure support actions: ranking based on the combination of the criteria *importance* and *achievability*

| # | Recommended action | Corresponding Food-EPI domain |
| --- | --- | --- |
| 1 | Demonstrate clear, knowledge-based, and coherent political leadership in public health and nutrition policies | LEADERSHIP |
| 2 | Ensure that there is access to qualified nutrition and food competence in the public sector. | FUNDING |
| 3 | Ensure that nutrition is strengthened as part of public health actions and that "health in all policies" is implemented at all levels. | HIAP |
| 4 | Monitor the compliance with the national Norwegian Guidelines for Food and Meals in schools, kindergartens, and after-school clubs, including in school canteens and kiosks. | MONITORING |
| 5 | Ensure long-term financing of effective and health promoting nutrition and public health work in counties and municipalities. | FUNDING |
| 6 | Integrate food and nutrition literacy as central parts of health literacy and obtain data on knowledge about nutrition in the population. | MONITORING |
| 7 | Ensure escalation of efforts around monitoring and data collection in terms of diet, nutrition, and health (regular dietary surveys, updating the food composition table, collect objectively measured data on overweight and obesity for adults, increase frequency of weighting children, national tool for risk markers for NCDs). | MONITORING |
| 8 | Ensure coordination and coherence between nutrition and all other relevant policy areas, in particular public health, agricultural, and fisheries policies, by establishing an inter-ministerial working group. | PLATFORMS |
| 9 | Ensure improved involvement of the voluntary sector and civil society in the nutrition policy | PLATFORMS |
| 10 | Contribute to protecting nutrition policy from undue influence from commercial interests by map the extent to which the food industry has influenced policy making, create a lobby register and use systems for handling conflict of interest in policy development | GOVERNANCE |
| 11 | Create the position of a Minister of Public Health under the Prime Minister's Office | LEADERSHIP |

Table S2-B – Recommended infrastructure support actions: ranking based on the criteria *importance*

| # | Recommended action | Corresponding Food-EPI domain |
| --- | --- | --- |
| 1 | Demonstrate clear, knowledge-based, and coherent political leadership in public health and nutrition policies | LEADERSHIP |
| 2 | Ensure that there is access to qualified nutrition and food competence in the public sector. | FUNDING |
| 3 | Ensure long-term financing of effective and health promoting nutrition and public health work in counties and municipalities. | FUNDING |
| 4 | Ensure that nutrition is strengthened as part of public health actions and that "health in all policies" is implemented at all levels. | HIAP |
| 5 | Ensure escalation of efforts around monitoring and data collection in terms of diet, nutrition, and health (regular dietary surveys, updating the food composition table, collect objectively measured data on overweight and obesity for adults, increase frequency of weighting children, national tool for risk markers for NCDs). | MONITORING |
| 6 | Monitor the compliance with the national Norwegian Guidelines for Food and Meals in schools, kindergartens, and after-school clubs, including in school canteens and kiosks. | MONITORING |
| 7 | Integrate food and nutrition literacy as central parts of health literacy and obtain data on knowledge about nutrition in the population. | MONITORING |
| 8 | Ensure coordination and coherence between nutrition and all other relevant policy areas, in particular public health, agricultural, and fisheries policies, by establishing an inter-ministerial working group. | PLATFORMS |
| 9 | Contribute to protecting nutrition policy from undue influence from commercial interests by map the extent to which the food industry has influenced policy making, create a lobby register and use systems for handling conflict of interest in policy development | GOVERNANCE |
| 10 | Ensure improved involvement of the voluntary sector and civil society in the nutrition policy | PLATFORMS |
| 11 | Create the position of a Minister of Public Health under the Prime Minister's Office | LEADERSHIP |

Table S2-C – Recommended infrastructure support actions: ranking based on the criteria *achievability*

| # | Recommended action | Corresponding Food-EPI domain |
| --- | --- | --- |
| 1 | Demonstrate clear, knowledge-based, and coherent political leadership in public health and nutrition policies | LEADERSHIP |
| 2 | Monitor the compliance with the national Norwegian Guidelines for Food and Meals in schools, kindergartens, and after-school clubs, including in school canteens and kiosks. | MONITORING |
| 3 | Integrate food and nutrition literacy as central parts of health literacy and obtain data on knowledge about nutrition in the population. | MONITORING |
| 4 | Ensure improved involvement of the voluntary sector and civil society in the nutrition policy | PLATFORMS |
| 5 | Ensure that nutrition is strengthened as part of public health actions and that "health in all policies" is implemented at all levels. | HIAP |
| 6 | Ensure that there is access to qualified nutrition and food competence in the public sector. | FUNDING |
| 7 | Ensure coordination and coherence between nutrition and all other relevant policy areas, in particular public health, agricultural, and fisheries policies, by establishing an inter-ministerial working group. | PLATFORMS |
| 8 | Create the position of a Minister of Public Health under the Prime Minister's Office | LEADERSHIP |
| 9 | Ensure escalation of efforts around monitoring and data collection in terms of diet, nutrition, and health (regular dietary surveys, updating the food composition table, collect objectively measured data on overweight and obesity for adults, increase frequency of weighting children, national tool for risk markers for NCDs). | MONITORING |
| 10 | Contribute to protecting nutrition policy from undue influence from commercial interests by map the extent to which the food industry has influenced policy making, create a lobby register and use systems for handling conflict of interest in policy development | GOVERNANCE |
| 11 | Ensure long-term financing of effective and health promoting nutrition and public health work in counties and municipalities. | FUNDING |
